# Procalcitonin-guided antibiotic therapy in intensive care unit patients: a systematic review and meta-analysis

**DOI:** 10.1186/s13613-017-0338-6

**Published:** 2017-11-22

**Authors:** Hui-Bin Huang, Jin-Min Peng, Li Weng, Chun-Yao Wang, Wei Jiang, Bin Du

**Affiliations:** 1 Medical ICU, Peking Union Medical College Hospital, Peking Union Medical College and Chinese Academy of Medical Sciences, 1 Shuai Fu Yuan, Beijing, 100730 People’s Republic of China; 2 Department of Critical Care Medicine, The First Affiliated Hospital of Fujian Medical University, Fuzhou, China

**Keywords:** Procalcitonin, Antibiotic strategies, Meta-analysis, Systematic review, Intensive care unit

## Abstract

**Background:**

Serum procalcitonin (PCT) concentration is used to guide antibiotic decisions in choice, timing, and duration of anti-infection therapy to avoid antibiotic overuse. Thus, we performed a systematic review and meta-analysis to seek evidence of different PCT-guided antimicrobial strategies for critically ill patients in terms of predefined clinical outcomes.

**Methods:**

We searched for relevant studies in PubMed, Embase, Web of Knowledge, and the Cochrane Library up to 25 February 2017. Randomized controlled trials (RCTs) were included if they reported data on any of the predefined outcomes in adult ICU patients managed with a PCT-guided algorithm or according to standard care. Results were expressed as risk ratio (RR) or mean difference (MD) with accompanying 95% confidence interval (CI).

**Data synthesis:**

We included 13 trials enrolling 5136 patients. These studies used PCT in three clinical strategies: initiation, discontinuation, or combination of antibiotic initiation and discontinuation strategies. Pooled analysis showed a PCT-guided antibiotic discontinuation strategy had fewer total days with antibiotics (MD − 1.66 days; 95% CI − 2.36 to − 0.96 days), longer antibiotic-free days (MD 2.26 days; 95% CI 1.40–3.12 days), and lower short-term mortality (RR 0.87; 95% CI 0.76–0.98), without adversely affecting other outcomes. Only few studies reported data on other PCT-guided strategies for antibiotic therapies, and the pooled results showed no benefit in the predefined outcomes.

**Conclusions:**

Our meta-analysis produced evidence that among all the PCT-based strategies, only using PCT for antibiotic discontinuation can reduce both antibiotic exposure and short-term mortality in a critical care setting.

**Electronic supplementary material:**

The online version of this article (10.1186/s13613-017-0338-6) contains supplementary material, which is available to authorized users.

## Background

Timely diagnosis and appropriate antimicrobial treatment of infection remain a major challenge in critical care settings. Delay in diagnosis due to lack of specific clinical signs in the early stage of infection may withhold or delay antibiotic therapy. On the other hand, concern of not treating potentially life-threatening infection and the risk of recurrence frequently leads clinicians to antimicrobial overuse in intensive care unit (ICU) [1, 2]. Studies have demonstrated that up to 50% of antibiotics prescribed in hospital settings are either unnecessary or inappropriate [3]. Nowadays, long-term antimicrobial regimens applied to critically ill patients are common and often based on empiric rules [4, 5]. This may result in increased medical costs, emergence of resistant pathogens, prolonged length of stay (LOS), and risk of mortality [6, 7].

Recently, procalcitonin (PCT) has shown to be a promising biomarker for identification of bacterial infections and is correlated with the severity of infection [8–11]. The 2016 Surviving Sepsis Campaign (SSC) guidelines offered a weak recommendation (low quality of evidence), favouring that measurement of procalcitonin levels can be used to support shortening the duration of antimicrobial therapy in sepsis patients [12]. However, one recent large study failed to show any benefit of daily PCT measurement with regard to time to appropriate therapy or survival, but resulted in a longer antibiotic course and ICU stay [13]. To date, several meta-analyses have assessed the value of PCT to guide antibiotic stewardship in ICU patients [14–22]. Findings of these reports showed that utilizing PCT to guide antibiotic decisions could significantly reduce antibiotics use, but did not improve patient outcomes, such as mortality, hospital, or ICU LOS. However, one major limitation of these meta-analyses was unexplainable significant heterogeneity among included trials, possibly due to the fact that the different PCT guidance strategies (including antibiotic initiation, discontinuation, or combination of antibiotic initiation and discontinuation strategies) had been evaluated in these trials. Since the considerable differences in methodologies and research purposes associated with the different PCT-guided strategies, the previous meta-analyses might not accurately evaluate the effects of PCT-based algorithms (Additional file 1: Table S1). Recently, two large-scale randomized controlled trials (RCTs) of PCT-guided antibiotic strategy in ICU patients have been published, with inconsistent results [23, 24]. Of note, the study by de Jong et al., the largest PCT trial to date, demonstrated an unexpected and significant survival benefit, in addition to less antibiotic exposure.

Therefore, with the aid of increased power of meta-analytic techniques, we sought to expand the previous analyses by including studies published recently, stratifying different strategies to have a more accurate analysis of the influence of different PCT algorithms to guide antimicrobial decisions.

## Methods

### Search strategy and selection criteria

This systematic review and meta-analysis were conducted in accordance with the PRISMA guidance [25]. We searched RCTs in PubMed, Embase, Web of Science, and Cochrane Central Register of Controlled Trials from inception through 25 February 2017 to identify potentially relevant studies. Search included the following key words: (“Procalcitonin” OR “PCT”) AND (“intensive care” OR “critically ill” OR “critical care”). No language restriction was imposed. Reference lists of relative articles were also reviewed.

Studies were included if they are enrolling adult ICU patients, with confirmed or suspected infection, assigned to either a PCT-guided therapeutic strategy group or a standard care group. Standard care referred to antimicrobial regimens based on clinical signs, laboratory results, and empiric rules or guidelines, without consideration of PCT level. We excluded studies enrolling children or patients without any evidence of infection and studies without mentioning of PCT assay methods. Articles available only in abstract form or meeting reports were also excluded.

### Data extraction and outcomes

Two reviewers independently extracted data from included studies on the first author, year of publication, country, sample size, study design, ICU type, compared protocols, methods of PCT assay, methodological quality, as well as all outcomes of interest.

We stratified different PCT-guided strategies according to medical decision with regards to antimicrobial therapy. In brief, strategy of antibiotic initiation referred to the decision to or not to start antibiotics, and decision of the intensified monitoring, diagnostic efforts, and interventions to explore uncontrolled sources of infection based on a predefined threshold of baseline PCT concentration, while strategy of antibiotic discontinuation meant making the decision to stop antibiotics according to a predefined threshold of PCT concentration, or the PCT level dropped by a certain proportion predefined compared with the previous value. The primary outcomes were the duration of antibiotic use and the short-term mortality, while the latter was defined as ICU or hospital or 28-day mortality [26, 27]. Secondary outcomes included ICU and hospital LOS.

### Quality assessment

Two independent reviewers evaluated the quality of studies using the risk of bias tool recommended by the Cochrane Collaboration [28]. We assigned a value of high, unclear, or low to the following items: sequence generation; allocation concealment; blinding; incomplete outcome data; selective outcome reporting; and other sources of bias. Discrepancies were identified and resolved through discussion.

### Statistical analysis

The results from all relevant studies were combined to estimate the pooled risk ratio (RR) and associated 95% confidence intervals (CIs) for dichotomous outcomes. As to the continuous outcomes, mean differences (MD) and 95% CI were estimated as the effect results. Heterogeneity was tested by using the *I* ^2^ statistic. An *I* ^2^ < 50% was considered to indicate insignificant heterogeneity, and a fixed-effect model was used, whereas a random-effect model was used in cases of significant heterogeneity (*I* ^2^ > 50%). Before data analysis, we estimated mean from median and standard deviations (SD) from IQR using the methods described in previous studies [29]. Sensitivity analyses were performed by excluding trials that potentially biased the results of primary outcomes. Publication bias was evaluated by visually inspecting funnel plots. All analyses were performed using Review Manager version 5.3.

## Results

### Study selection

**Fig. 1:**
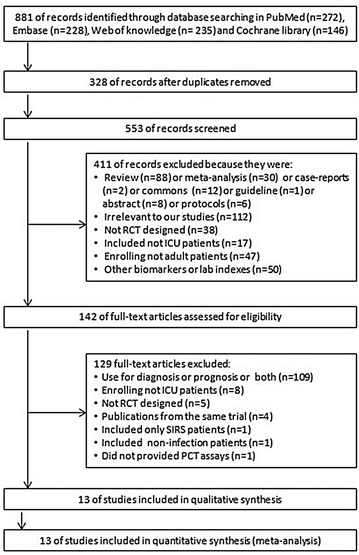
Selection process for RCTs included in the meta-analysis

The literature search yielded 881 records through database searching, and 13 RCTs fulfilled inclusion criteria were eligible for the final analysis [13, 23, 24, 30–39]. The overview of the study selection process is presented in Fig. 1. In the study by Jensen et al., some patients without infection were also included [13]; therefore, we only included patients with severe sepsis or septic shock that fulfilled our inclusion criteria from this study. The Cochrane risk of bias score for each citation varied across the studies (Additional file 2: Table S2).

### Study characteristics

The main characteristics and predefined outcomes of the 13 included studies are shown in Table 1 and Additional file 3: Table S3. The degree of non-compliance with PCT algorithm recommendations for antibiotics varied among the included RCTs (Additional file 4: Table S4). Of these included studies, seven were multicenter studies. A total of 5136 patients comprised 2588 in the PCT-guided group and 2548 in the standard care group. These studies evaluated the effects of PCT-guided strategies in antibiotic discontinuation (*n* = 8) [23, 24, 31–34, 37, 39], antibiotic initiation (*n* = 3) [13, 30, 36], or the combination of the antibiotic initiation and discontinuation (*n* = 2) [35, 38]. Study population included surgical patients (*n* = 3) [30, 31, 33] and mixed medical–surgical patients (*n* = 10) [13, 23, 24, 32, 34–39]. PCT assays adopted varied across the included studies.

**Table 1:** Characteristics of included studies

Study/year	Trial design	Population	Type of ICU	*N* PCT/Ctrl	PCT-guided group protocol	Control group protocol	PCT assay
Svoboda et al. [30]	SC, P, R, OL	Postoperative severe sepsis	Surgical	38/34	AI: prompted change of ABT and catheter (≥ 2 ng/ml), prompted to repeated radiographic and/or surgical evaluation (< 2 ng/ml)	Standard evaluation by consultant surgeon	PCT-Q
Schroeder et al. [31]	SC, P, R, OL	Postoperative severe sepsis	Surgical	14/13	AD: if clinic signs and symptoms improved and PCT < 1 ng/ml or 25–35% of baseline	According to clinical signs and empiric rules	LIA
Nobre et al. [32]	SC, P, R, OL	Sepsis	Mixed	39/40	AD: if baseline PCT > 1 μg/L, re-evaluation at day 5. ABT discontinuation if PCT < 0.25 μg/L or PCT dropped by > 90% from the baseline peak level. If baseline PCT < 1 μg/L, re-evaluation at day 3. ABT discontinuation if PCT < 0.1 μg/L and careful clinical evaluation	Regimens according to guidelines	Kryptor
Hochreiter et al. [33]	SC, P, R, OL	Infection	Surgical	53/57	AD: if clinic signs and symptoms improved and PCT < 1 ng/ml or 25–35% of initial value over 3 days	Standard regimen over 8 days	LIA
Stolz et al. [34]	MC, P, R, OL	Ventilator-associated pneumonia	Mixed	51/50	AD: strongly encouraged (< 0.25 μg/L), encouraged (0.25–0.5 μg/L or a decrease ≥ 80%), discouraged (0.5–1.0 μg/L or a decrease < 80%) or strongly discouraged (> 1.0 μg/L)	According to clinical signs and empiric rules	Kryptor
Bouadma et al. [35]	MC, P, R, OL	Bacterial infection or sepsis	Mixed	311/319	AI: ABT was strongly discouraged (< 0.25 μg/L), discouraged (0.25–0.49 μg/L), encouraged (0.5–0.99 μg/L) or strongly encouraged (≥ 1 μg/L) AD: strongly encouraged (< 0.25 μg/L), encouraged (0.25–0.49 μg/L). continuing of ABT was encouraged (0.25–0.5 μg/L or > 80% peak) and change of ABT (> peak concentration and > 0.5 μg/L)	Regimens according to international and local guidelines	Kryptor
Jensen et al. [13]	MC, P, R, OL	Severe sepsis/septic shock	Mixed	212/247	AI: if PCT ≥ 1 μg/L that was not decreasing by at least 10% from previous day: increasing the antimicrobial spectrum and intensifying diagnostic efforts to find uncontrolled sources of infection	According to current guidelines	Kryptor
Layios et al. [36]	SC, P, R, OL	Infection	Mixed	258/251	AI: ABT was strongly discouraged (< 0.25 μg/L), discouraged (0.25–0.5 μg/L), encouraged (0.5–1.0 μg/L) or strongly encouraged (> 1.0 μg/L)	No reports	Kryptor
Annane et al. [38]	MC, P, R, OL	Septic shock	Mixed	31/31	AI/AD: ABT was not to be started or was to be discontinuation (< 0.25 μg/L); strongly discouraged (≥ 0.25 to < 0.5 μg/L); was recommended (≥ 0.5 to < 5 μg/L) and was strongly recommended (≥ 5 μg/L). For patients enrolled ≤ 48 h after surgery, the respective PCT cut-offs were < 4 μg/L, 4–9 μg/L and ≥ 9 μg/L	ABT at the discretion of the patient’s physician	Kryptor
Deliberato et al. [39]	SC, P, R, OL	Sepsis	Mixed	42/39	AD: if PCT dropped > 90% from the peak level or the absolute value < 0.5 ng/ml	The possible source of infection and local susceptibility profile	Vidas
Shehabi et al. [23]	MC, P, R, SB	Bacterial infection or sepsis	Mixed	200/200	AD: cease ABT when PCT < 0.1 ng/ml or PCT was 0.1–0.25 ng/ml and infection is highly unlikely or PCT level decreased > 90% from baseline	According to the ABT guidelines	Automated immunoassay analysers
De Jong et al. [24]	MC, P, R, OL	Infection	Mixed	776/799	AD: if PCT value decreased over 80% or PCT value lower than 0.5 μg/L	Guidelines and the discretion of attending physicians	Vidas, Roche or Kryptor machine
Bloos et al. [37]	MC, P, R, OL	Severe sepsis/septic shock	Mixed	587/593	AD: stopping ABT if PCT level on day 7 or later < 1 ng/ml r or dropped > 50% from the previous value	According to the local sepsis guidelines	Kryptor

*ABT* antibiotics, *AD* antibiotic discontinuation, *AI* antibiotic initiation, *Ctrl* control, *ICU* intensive care unit, *LIA* immunoluminometric assay, *MC* multi-centre, *Mixed* surgical and medical intensive care unit, *OL* open label, *P* prospective, *PCT* procalcitonin, *PCT-Q* procalcitonin immunochromatographic technology, *R* RCT, *SC* single centre

### Data synthesis

#### Procalcitonin-guided discontinuation of antibiotics

**Fig. 2:**
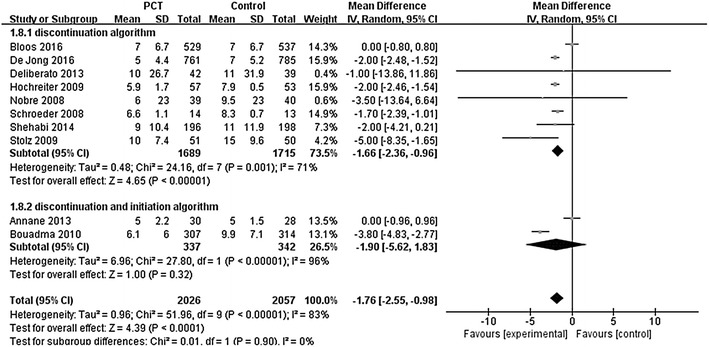
Effects of PCT-guided antimicrobial strategies on total days of antibiotics

**Fig. 3:**
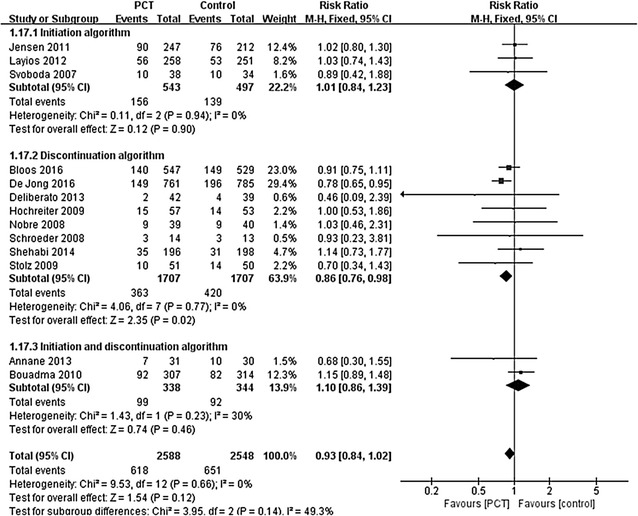
Effects PCT-guided antimicrobial strategies on short-term mortality

The use of a PCT algorithm compared with standard care to guide antibiotic discontinuation in critically ill patients was evaluated in eight RCTs [23, 24, 31–34, 37, 39]. All eight studies reported outcomes including total days with antibiotics or antibiotic-free days. The aggregated data suggested that the duration of antibiotic treatment was 1.67 days shorter in PCT-guided group (*n* = 3404; MD − 1.66 days; 95% CI − 2.36 to − 0.96; *I* ^2^ = 71%; *P* < 0.00001) [23, 24, 31–34, 37, 39] (Fig. 2), while antibiotic-free days were 2.26 days longer (*n* = 2120; MD 2.26 days; 95% CI 1.40–3.12; *I* ^2^ = 0%; *P* < 0.00001) [23, 24, 32, 34] when compared with that of standard care group. Results showed patients in PCT-guided group had lower short-term mortality than standard care group (*n* = 3414; RR 0.86; 95% CI 0.76–0.98; *I* ^2^ = 0%; *P* = 0.02) [23, 24, 31–34, 37, 39] (Fig. 3), while no differences were found in ICU LOS (*n* = 3326; MD − 0.00 days; 95% CI − 0.58 to 0.58; *I* ^2^ = 0%; *P* = 0.99) [23, 24, 31–33, 37, 39] and hospital LOS (*n* = 3290; MD 0.43 days; 95% CI − 0.83 to 1.70; *I* ^2^ = 30.4%; *P* = 0.50) [23, 24, 32, 34, 37, 39]. There was significant heterogeneity in the outcome of duration of antibiotic treatment between the pooled studies. Therefore, we conducted sensitivity analyses to explore potential sources of heterogeneity. Exclusion of the trial by Bloos and colleagues resolved the heterogeneity without alerting the result (*n* = 2338; MD − 1.97 days; 95% CI − 2.27 to − 1.68; *I* ^2^ = 0%; *P* < 0.00001) [24, 31–34, 37, 39].

#### Procalcitonin-guided initiation of antibiotics

Three studies examined the efficacy of PCT-guided initiation of antibiotics [13, 30, 36]. Only one study examined the efficacy of PCT-guided initiation of antibiotics. In this case, antibiotic consumption was comparable between groups with the treatment days represented 62.6% and 57.7% of ICU stays in the PCT and standard care groups, respectively (*P* = 0.11) [36]. There was no statistically significant difference between groups in the risk of short-term mortality (*n* = 1040; RR 1.01; 95% CI 0.84–1.23; *I* ^2^ = 0%; *P* = 0.90) [13, 30, 36] (Fig. 3) or ICU LOS (*n* = 581; MD − 1.22 days; 95% CI − 4.34 to 1.90; *I* ^2^ = 60%; *P* = 0.44) [30, 36].

#### Procalcitonin-guided antibiotic initiation and discontinuation

Two studies employed a PCT-guided strategy of antibiotic initiation and discontinuation [35, 38]. No differences were observed between the PCT and standard care group in total days with antibiotics (*n* = 679; MD − 1.90 days, 95% CI − 5.62 to 1.83; *I* ^2^ = 96%; *P* = 0.32) (Fig. 2), antibiotic-free days (*n* = 679; MD 1.31 days; 95% CI − 1.34 to 3.95; *I* ^2^ = 90%; *P* = 0.33), short-term mortality (*n* = 682; RR 1.10; 95% CI 0.86–1.39; *I* ^2^ = 30%; *P* = 0.46) (Fig. 3), the ICU LOS (*n* = 682; MD − 1.45 days; 95% CI − 0.91 to 3.80; *I* ^2^ = 0%; *P* = 0.23), and hospital LOS (*n* = 679; MD − 0.43 days; 95% CI − 3.36 to 2.49; *I* ^2^ = 0%; *P* = 0.77) [35, 38].

Summary of findings for the effect of PCT-guided strategy antibiotic on predefined outcomes in ICU patients is described in Table 2. We did not assess the publication bias because of the limited number of studies included in each analysis.

**Table 2:** Summary of findings for the effect of procalcitonin-guided strategy on predefined outcomes in intensive care unit patients

PCT-guided strategy	Predefined outcome	Number of trials	*N*	Estimated benefit with antibiotic	*I* ^2^ (%)	*P* value
ABT discontinuation	Duration of antibiotic use	8	3404	− 1.66 days (− 2.36, − 0.96)	71	*P* < 0.0001
	Antibiotic-free days	4	2120	2.26 days (1.40, 3.12)	0	*P* < 0.0001
	Short-term mortality	8	3414	0.86 (0.76, 0.98)	0	0.02
	Length of stay in ICU	7	3326	− 0.00 days (− 0.58, 0.58)	0	0.99
	Length of stay in hospital	6	3290	0.43 days (− 0.83, 1.70)	30	0.50
ABT initiation	Duration of antibiotic use	–	–	–	–	–
	Antibiotic-free days	–	–	–	–	–
	Short-term mortality	3	1040	1.01 (0.84, 1.23)	0	0.90
	Length of stay in ICU	2	581	− 1.22 days (− 4.34, 1.90)	60	0.44
	Length of stay in hospital	–	–	–	–	–
ABT initiation and discontinuation	Duration of antibiotic use	2	679	− 1.90 days (− 5.62, 1.83)	96	0.32
	Antibiotic-free days	2	679	1.31 days (− 1.34, 3.95)	90	0.33
	Short-term mortality	2	682	1.10 (0.86, 1.39)	30	0.46
	Length of stay in ICU	2	682	− 1.45 days (− 0.91, 3.80)	0	0.23
	Length of stay in hospital	2	750	− 0.43 days (− 3.36, 2.49)	0	0.77

*ABT* antibiotics, *PCT* procalcitonin, *ICU* intensive care unit

## Discussion

PCT-guided strategies had been examined in multiple studies to optimize antibiotic treatment, with conflicting results. The current meta-analysis justified a low PCT level to discontinue antibiotic treatment, which would result in a shorter duration of antibiotic treatment of about 1.67 days, as well as lower short-term mortality compared with standard care. Due to the insufficient evidence, a baseline PCT value should not be used as a marker to guide antibiotic initiation.

Our study had several strengths. The current meta-analysis provided robust evidence to support and expand the weak suggestion in the 2016 SSC guidelines, i.e. use of low PCT level to assist the clinician in the discontinuation of empiric antibiotic [12]. In addition to those RCTs in previous meta-analyses, we had included three additional RCTs recently published, and this added to the statistical power by having 3414 cases to evaluate the primary outcome. Moreover, we had stratified enrolled RCTs according to different PCT-guided strategies, in order to eliminate the potential confounding factors caused by different strategies. Though significant heterogeneity was observed among these studies, our sensitivity analyses demonstrated that the heterogeneity was resulted from the trial by Bloss et al. [23]. This study was different from the other trials in some aspects. On the one hand, it was designed as a 2 × 2 factorial trial and the interaction between the two treatment factors was unclear; on the other hand, in the trial [23], the clinicians used a 50% decrease from previous value as a stopping rule, which was lower than that of other studies. As a result, we also demonstrated a significant improvement in short-term mortality associated with PCT-guided antibiotic discontinuation. This had added robustness to findings of reduction in antibiotics usage, since studies have demonstrated that strategies aiming at restricting antibiotic overuse could help improve survival [40, 41]. Our findings that short-term mortality was significantly reduced in the PCT-guided discontinuation group contrasted those of previous meta-analyses. Despite the fact that no heterogeneity was detected, the beneficial effect was clearly driven by the study results of de Jong et al. [24]. However, as the authors acknowledged, this study was a non-inferiority study; therefore, the beneficial effect of mortality in PCT-guided group was unexpected, which merited cautious interpretation and further validation.

As for a PCT strategy that combined initiation and discontinuation of antibiotics, we found no beneficial effect with regard to any predefined outcomes. The reason for this failure may be that only two trials (one positive [35] and one negative [38]) investigated the combined strategies varying in objective and the methodology. On the other hand, reported non-compliance rate was high. For example, in a prospective, multicenter, open-label randomized trial (PRORATA trial) involving 630 non-surgical patients with suspected bacterial infections [35], patients in the PCT group had significantly more antibiotic-free days. However, the algorithm-guided treatment recommendation was not strictly followed in 53% of patients in the PCT group. Moreover, this trial [35] reported a higher standard deviation with regard to duration of antibiotic treatment as well as antibiotic-free days (possibly due to reported higher non-compliance rate), compared to that in the negative trial [38], which weaken its statistic weight in the meta-analysis. Interestingly, it was noteworthy that initial antibiotic prescription rate was similar in PCT and standard care groups, suggesting that the improvement in antibiotic-free days was more likely the result of antibiotic discontinuation, while it was less likely due to exclusion of potential infection.

In our study, we could not verify the efficacy of PCT-based antibiotic initiation strategy because we found only three RCT through the literature search [13, 30, 36]. Of these trials, only the trial by Layios and coworkers reported the antibiotics exposure and concluded that PCT measuring for the initiation of antibiotics failed to decrease the antibiotic consummation [36]. The reasons for this failure may be that almost half of PCT serum samples were > 1 μg/L, thus encouraging the antibiotic treatment, and the relative low proportion of patient-days with antibiotic treatment in the control group (57%). Another reason could be related to the high non-compliance rate with the PCT-guided antibiotic initiation strategy described in the study. The authors reported that nearly 64% of patients in the PCT group received antibiotics regardless of a normal PCT level (< 0.5 μg/L).

The incidence of non-compliance with the recommendations based on PCT algorithm, as reported in some, but not all RCTs, showed significant variability, ranging from 0 to 59%. In most case of non-compliance, physicians were reluctant to stop antibiotics, even with a very low PCT level (Additional file 4: Table S4), possibly due to the concerns about the accuracy of single PCT value as a biomarker of infection [42, 43]. This might lead to unnecessarily prolonged exposure of antibiotics, which supported the robustness of our findings that implementation of PCT algorithm was associated with shorter duration of antibiotic treatment and longer antibiotic-free days.

Our meta-analysis has some limitations. First, studies examining the PCT-guided strategies other than discontinuation of antibiotic treatment were scarce, with limited number of studies available as well as small number of enrolled patients. As such, data on these strategies were insufficient to draw solid conclusions. Second, the high exclusion rate of screened patients in the included studies (such as immunosuppressed patients and those requiring long-term antibiotic therapy) precluded generalization of the study results. Third, antibiotic strategy in the control group (indications to initiate and discontinue antibiotics) was not specified in most studies. Whether the variation in antibiotic strategy, if any, in the control group might have affected the results of our meta-analysis is unclear. Thus, a more uniform approach to evaluating and reporting standard care related to antibiotic use would be needed in future studies. Fourth, the uneven distribution of different underlying diseases among included studies might also exert a prognostic value. Of note, in the two recently published systematic reviews and individual patient data meta-analysis [44, 45], Schuetz et al. demonstrated with sufficient evidence that PCT-guided antibiotic treatment in patients with acute respiratory infections reduced antibiotic exposure and side effects and improved survival. Finally, different cut-off values of PCT and different PCT measurements were reported across included studies, which might also lead to bias in our results. We had originally tried to perform subgroup analyses exploring studies according to all the diversities. However, there were insufficient data.

## Conclusions

In summary, based on the results of our meta-analysis, we recommend use of PCT to guide antibiotic discontinuation, which was associated with a reduction in antibiotic exposure and lower short-term mortality. Further studies are needed to define the optimal cut-off value of PCT for antibiotic discontinuation and to generalize our findings in other patient population including immunocompromised patients and those received long-term antibiotic therapy in ICU.

## Additional files

**Additional file 1: Table S1.** Summary of the RCTs included by previous and current meta-analysis.

**Additional file 2: Table S2.** Predefined outcomes of the included RCTs.

**Additional file 3: Table S3.** Risk of bias table for included randomized control trials.

**Additional file 4: Table S4.** Non-compliance rate reported in the included RCTs.

## Abbreviations

CI: confidence interval
ICU: intensive care unit
IQR: interquartile range
LOS: length of stay
MD: mean difference
PCT: procalcitonin
RR: risk ratio
RCTs: randomized controlled trials
SD: standard deviations
VAP: ventilator-associated pneumonia
