# Supplementary material for: Procalcitonin-guided antibiotic therapy in intensive care unit patients: a systematic review and meta-analysis
Source: Ann Intensive Care. 2017 Nov 22;7:114. doi: 10.1186/s13613-017-0338-6 (PMC5700008; doi:10.1186/s13613-017-0338-6)
Supplement: Supplementary file 1 — Additional file 1: Table S1. Summary of the RCTs included by previous and current meta-analysis. [file 13613_2017_338_MOESM1_ESM.docx]

**Additional file 1: Table S1. Summary of the RCTs included by previous and current meta-analysis**

| Study / Year | Sample  size | Tang  2009* | Kopterides  2010† | Heyland 2011 | Schuetz  2011 | Agarwal 2011 | Soni  2012 | Matthaiou  2012 | Prkno  2013 | Westwood  2015 | Current  Review |
| --- | --- | --- | --- | --- | --- | --- | --- | --- | --- | --- | --- |
| Algorithms for intensification of antibiotic therapy | | | | | | | | | | | |
| Layios 2012 | 509 |  |  |  |  |  |  |  |  | √ | √ |
| Algorithms for initiation of antibiotic therapy | | | | | | | | | | | |
| Svoboda 2007 | 72 | √ | √ |  | √ |  | √ | √ | √ |  | √ |
| Layios 2009§ | 529 |  |  |  |  | √ |  |  |  |  |  |
| Jensen 2011 | 1200 |  |  |  |  |  | √ | √ | √‡ |  | √‡ |
| Algorithms for discontinuation of antibiotic therapy | | | | | | | | | | | |
| Nobre 2008 | 79 | √ | √ | √ | √ | √ | √ | √ | √ | √ | √ |
| Hochreiter 2009 | 110 | √ | √ | √ | √ | √ | √ | √ | √ |  | √ |
| Stolz 2009 | 101 |  | √ | √ | √ | √ | √ | √ |  | √ | √ |
| Schroeder 2009 | 27 |  | √ | √ | √ | √ | √ | √ | √ |  | √ |
| Liu 2013^\|\|^ | 82 |  |  |  |  |  |  |  |  | √ |  |
| Deliberato 2013 | 81 |  |  |  |  |  |  |  |  | √ | √ |
| Shehabi 2014 | 400 |  |  |  |  |  |  |  |  |  | √ |
| De Jong 2016 | 1575 |  |  |  |  |  |  |  |  |  | √ |
| Bloss et al 2016 | 1089 |  |  |  |  |  |  |  |  |  | √ |
| Algorithms for initiation and discontinuation of antibiotic therapy | | | | | | | | | | | |
| Bouadma 2010 | 630 |  | √ | √ | √ | √ | √ | √ | √ | √ | √ |
| Qu 2012 | 71 |  |  |  |  |  |  |  |  | √ |  |
| Annane 2013 | 62 |  |  |  |  |  |  |  | √ | √ | √ |

*The review by Tang et al also included other 4 RCT that enrolled non-ICU patients. † The review by Kopterides et al also included 1 RCT that enrolled neonates. ‡Only the patients with severe sepsis and

septic shock from the study conducted by Jensen et al. were included. §The trial by Layios et al was only in abstract form.
