# Supplementary material for: Procalcitonin-guided antibiotic therapy in intensive care unit patients: a systematic review and meta-analysis
Source: Ann Intensive Care. 2017 Nov 22;7:114. doi: 10.1186/s13613-017-0338-6 (PMC5700008; doi:10.1186/s13613-017-0338-6)
Supplement: Supplementary file 2 — Additional file 2: Table S2. Predefined outcomes of the included RCTs. [file 13613_2017_338_MOESM2_ESM.docx]

**Additional file 2: Table S2. Predefined outcomes of the included RCTs**

| Author  year | N  PCT / Ctrl | Mortality (PCT / Ctrl) | | | | LOS in ICU | LOS in hospital | Duration of antibiotic use | Antibiotic-free days |
| --- | --- | --- | --- | --- | --- | --- | --- | --- | --- |
|  |  | **28-day** | **ICU** | **Hospital** | **Others** | **PCT-guide group / Ctrl group (Median (IQR) or mean (SD), Days)** | | | |
| Svoboda et al  2007 | 38/34 | 10/38  10/34 | - | - | - | 16.1 (6.9)  19.4 (8.9) | - | - | - |
| Schroeder et al  2008 | 14/13 | NR | - | 3/14  3/13 | - | 16.4 (8.3)  16.7 (5.6) | - | 6.6 (1.1)  8.3 (0.7) | - |
| Nobre et al  2008 | 39/40 | 8/39  8/40 | - | 9/39  9/40 | - | 4 (1 to 21)  7 (1 to 91) | 17 (3 to 96)  23.5 (5 to 44) | 6 (3 to 34)  9.5 (2 to 33) | 15.3 (8.9)  13 (8.2) |
| Hochreiter et al  2009 | 57/53 | NR | - | 15/57  14/53 | - | 17.7 (10.1)  15.5 (12.5) | - | 5.9 (1.7)  7.9 (0.5) | - |
| Stolz et al  2009 | 51/50 | 8/51  12/50 | - | 10/51  14/50 | - | - | 26 (7 to 21)  26 (16.8 to 22.3) | 10 (6 to 16)  15 (10 to 23) | 13 (2 to 21)  9.5 (1.5 to 17) |
| Bouadma et al  2010 | 311/319 | 65/307 64/314 | - | - | 92/307^*^  82/314^*^ | 15.9 (16.1)  14.4 (14.1) | 26.1 (19.3)  26.4 (18.3) | 6.1 (6)  9.9 (7.1) | 14.3 (9.1)  11.6 (8.2) |
| Jensen et al  2011 | 247/212 | 90/247  76/212 | - | - | - | - | - | - | - |
| Layios et al  2012 | 258/251 | - | 56/258 53/251 | - | - | 7 (4 to 16)  7 (4 to 18) | - | - | - |
| Annane et al  2013 | 31/31 | - | 7/31  10/30 | 7/31  10/30 | - | 22 (8 to 42)  23 (10 to 60) | 27 (9 to 49)  33 (11 to 69) | 5 (2 to 5)  5 (3 to 5) | 0 (0–3)  0 (0– 2) |
| Deliberato et al  2013 | 42/39 | - | 1/42  4/39 | 2/42  4/39 | - | 3.5 (1 to 57)  3 (1 to 28) | 11 (3 to 547)  11 (2, 228) | 10 (3 to 39)  11 (2 to 45) | - |
| Shehabi et al  2014 | 200/200 | - | 21/196  15/198 | 30/196  26/198 | 35/196^†^  31/198^†^ | 6 (3 to 9.5)  6 (4 to 10) | 15 (9 to 29)  17 (10 to 32) | 9 (6 to 20)  11 (6 to 22) | 20 (11 to 22)  17 (7 to 22) |
| De Jong et al  2016 | 776/799 | 149/761  196/785 | - | - | 256/761^‡^  321/785^‡^ | 8.5 (5.0 to 17)  9 (4.0 to 17) | 22 (13 to 39.3)  22 (12 to 40) | 5 (3 to 9)  7 (4 to 11) | 7 (0 to 14.5)  5 (0 to 13) |
| Bloss et al  2016 | 552/537 | 140/547  149/549 | - | - | 200/529^†^  199/516^†^ | 12 (6 to 24)  11 (6 to 21) | 29 (18 to 46)  26 (16 to 44) | 7 (3 to 12)  7 (3 to 12) | - |

* 60-Day mortality. † 90-Day mortality. ‡ 1-Year mortality. IQR= inter-quartile range. PCT=procalcitonin. NR=no reported. LOS=length of stay. Ctrl=control.
