# Supplementary material for: Procalcitonin-guided antibiotic therapy in intensive care unit patients: a systematic review and meta-analysis
Source: Ann Intensive Care. 2017 Nov 22;7:114. doi: 10.1186/s13613-017-0338-6 (PMC5700008; doi:10.1186/s13613-017-0338-6)
Supplement: Supplementary file 3 — Additional file 3: Table S3. Risk of bias table for included randomized control trials. [file 13613_2017_338_MOESM3_ESM.docx]

**Additional file 3: Table S3. Risk of bias table for included randomized control trials**

| First Author/ year | Random Sequence  Generation | Allocation  Concealment | Blinding of  Participants and Personnel | Blinding of  Outcome  Assessment | Incomplete  Outcome Data | Selective  Reporting | Other Bias |
| --- | --- | --- | --- | --- | --- | --- | --- |
| Svoboda 2007 | Low | Low | Unclear | Unclear | Low | Low | Low |
| Nobre 2008 | Low | Low | Unclear | Unclear | Unclear | Low | Low |
| Hochreiter 2009 | Unclear | Unclear | Low | Unclear | Low | Low | Low |
| Stolz 2009 | Unclear | Low | Unclear | Unclear | Low | Low | Low |
| Schroeder 2009 | Unclear | Unclear | Low | Unclear | Low | Unclear | Low |
| Bouadma 2010 | Low | Low | Unclear | Unclear | Low | Low | Low |
| Jensen 2011 | Low | Low | Low | Low | Low | High | Low |
| Layios 2012 | Unclear | Unclear | Unclear | Unclear | Low | Low | Low |
| Annane 2013 | Low | Unclear | Low | Low | Low | Low | Low |
| Deliberato 2013 | Unclear | Unclear | Unclear | Low | High | Low | Low |
| Shehabi 2014 | Low | Unclear | Unclear | Unclear | Low | Low | Unclear |
| De Jong 2016 | Low | Low | Low | Low | Low | Low | Low |
| Bloos 2016 | Low | Low | High | Unclear | Low | Low | Low |
