# Supplementary material for: Procalcitonin-guided antibiotic therapy in intensive care unit patients: a systematic review and meta-analysis
Source: Ann Intensive Care. 2017 Nov 22;7:114. doi: 10.1186/s13613-017-0338-6 (PMC5700008; doi:10.1186/s13613-017-0338-6)
Supplement: Supplementary file 4 — Additional file 4: Table S4. Non-compliance rate reported in the included RCTs. [file 13613_2017_338_MOESM4_ESM.docx]

**Additional file 4: Table S4. Non-compliance rate reported in the included RCTs**

| Study/Year | Rate | Described in the article |
| --- | --- | --- |
| Hochriter 2009 | NA |  |
| Schroeder 2008 | NA |  |
| Svoboda 2007 | NA |  |
| Layios 2012 | 34.6% | See Table 5.  “Only 36% of clinicians were compliant with the recommendation to withhold antimicrobials. This is in contrast to those who had a baseline PCT level >0.5μg/L, where 86% of clinicians were compliant with the recommendation to initiate antimicrobials. (Comment from other Review)  154/179=0.86; 46/127=0.36;  Total :(16+9+38+43)/306=34.6 |
| Stolz 2009 | 16% | “In the procalcitonin group, a total of 8 patients with improved CPIS scores received prolonged antibiotic treatment despite low procalcitonin values. In four (8%) cases, positive blood cultures with Gram negative bacilli were observed and in another four (8%) cases, treating physicians decided to prolong antibiotic treatment due to documented pulmonary infection with Gram negative bacilli”. (Result section)  (8/51=0.16) |
| Nobre 2008 | 19% | “Algorithm overruling’’ in the PCT group (i.e., treating physician refused to stop the antibiotics, although the stopping rules allowed this) occurred in 6 of 31 (19%) patients of the PCT group” (Result section)  (6/31=0.19) |
| Bouadma 2010 | 53% | “Recommendations about duration of antimicrobial treatment for the procalcitonin group were not followed in 219 episodes…”(Result section)  “Of the 219 episodes in which the procalcitonin algorithm was not followed, the algorithm was overruled at inclusion and during follow-up for 57 patients, and therefore the algorithm was not adhered to in 162 patients, corresponding to 53% of the procalcitonin group.” (Result section)  (219-57)/307=0.53； |
| Jensen 2011 | 17.9% | “In the procalcitonin group, 256 of 312 (82.1%) of patients with baseline “alert procalcitonin” received antimicrobials according to the available procalcitonin measurement and the intervention algorithm…”(Result section)  (1-0.821=0.179) |
| Annane 2013 | 37% | “In the experimental arm, physicians were noncompliant with the PCT-based algorithm in 19% of patients at 6 h, 17% on day 3 and 37% on day 5.(See Table 5)” |
| Deliberato 2013 | 0% | “Regarding the 20 patients in the PCT group (per-protocol analysis), 7 patients (35%) had their antibiotic therapy stopped based on the PCT <0.5 ng/mL, 5 patients (25%) based on a PCT drop >90%, and 8 (40%) had both criteria to discontinue antibiotic therapy.” (Result section)  PP:(1-0.35-0.25-0.4=0) |
| Shehabi 2014 | 3%? | “proportion of study days where the PCT algorithm was not followed was less than 3%, the majority of which was due to missed PCT sampling.” (Result section) |
| De Jong 2016 | 59% | “Adherence to this stopping advice was for 243 patients (44%) who had their antibiotic treatments stopped within 24 h and 297 patients (53%) treatments were stopped within 48 h after reaching the stopping threshold. 17 patients (3%) did not have their antibiotics stopped….” (Result section)  “Of the patients in whom physicians adhered to one of the stopping rules, 126 (42%) of 297 patients were stopped because of a decrease in procalcitonin concentrations to 20% or lower of the peak value, 154 (52%) of 297 patients were stopped as the procalcitonin concentration was 0·5 μg/L or lower, and 17 (6%) of 297 patients reached both these stopping rules simultaneously.” (Result section)  “Second, physicians did not adhere to the stopping advice in more than half of the patients.  (Discussion section, limitation part)  (56%+3%=59%) |
| Bloos 2016 | **59.1%** | “Adherence to the recommendation of the algorithm dropped to 40.9% commencing by day 7 (eTable8 in Supplement 2).”  (1-0.409=0.591) |
